# Different luminance- and texture-defined contrast sensitivity profiles for school-aged children

**DOI:** 10.1038/s41598-020-69802-5

**Published:** 2020-08-03

**Authors:** Daphné Silvestre, Jacalyn Guy, Julie Hanck, Kim Cornish, Armando Bertone

**Affiliations:** 1 Perceptual Neuroscience Lab (PNLab) for Autism and Development, Department of Education and Counselling Psychology, McGill University, 3700 McTavish Street, Montreal, QC H3A 1Y2 Canada; 2 MRC Cognition and Brain Sciences Unit, University of Cambridge, Cambridge, UK; 3 Integrated Program in Neuroscience, McGill University, Montreal, Canada; 4 Turner Institute for Brain and Mental Health, Monash University, Melbourne, Australia

**Keywords:** Perception, Human behaviour

## Abstract

Our current understanding of how the visual brain develops is based largely on the study of luminance-defined information processing. This approach, however, is somewhat limiting, since everyday scenes are composed of complex images, consisting of information characterized by physical attributes relating to both luminance and texture. Few studies have explored how contrast sensitivity to texture-defined information develops, particularly throughout the school-aged years. The current study investigated how contrast sensitivity to luminance- (luminance-modulated noise) and texture-defined (contrast-modulated noise) static gratings develops in school-aged children. Contrast sensitivity functions identified distinct profiles for luminance- and texture-defined gratings across spatial frequencies (SFs) and age. Sensitivity to luminance-defined gratings reached maturity in childhood by the ages of 9–10 years for all SFs (0.5, 1, 2, 4 and 8 cycles/degree or cpd). Sensitivity to texture-defined gratings reached maturity at 5–6 years for low SFs and 7–8 years for high SFs (i.e., 4 cpd). These results establish that the processing of luminance- and texture-defined information develop differently as a function of SF and age.

## Introduction

Everyday scenes are composed of different types of information that the visual system must identify, differentiate and organize into meaningful percepts. For instance, the visual system must parse these scenes into foreground and background elements, which may be defined by either luminance- (or first-order) or texture-defined (or second-order) information^1–3^. A considerable body of work suggests that different mechanisms process luminance- and texture-defined information; luminance is processed at earlier stages of analysis by linear filters in the primary visual cortex^4,5^, whereas texture is processed by non-linear filters in higher centers of the visual system^1,6–8^.

Traditional methods for understanding sensitivities to luminance- and texture-defined information include psychophysics, electrophysiology and neuroimaging^9–14^. Of these techniques, psychophysics is the most common, mainly for its simplicity and usefulness in generating contrast sensitivity functions^15–17^. This approach has led to a significant literature mostly motivated by understanding basic mechanisms rather than development^18–21^. Consequently, most of this research has focussed on adults and not children.

Despite this emphasis on adult findings, there has been considerable interest in understanding how basic visual functions develop early in life, specifically with respect to luminance-defined information^22–24^. Relative to adults, spatial contrast sensitivity for luminance-defined information is immature in infants^25–28^ and remains so early into childhood^29–34^. Beyond the age of 4 years, however, contrast sensitivity to luminance reaches maturity anywhere from 6 to 19 years^35^.

Unlike for luminance-defined information, few studies have examined the development of texture-defined information, especially using static stimuli^9,36^. Of these studies, most have focused on sensitivities to single spatial frequency (SF) or the differences between sensitivities to luminance- and texture-defined gratings of one SF^9,36^. To date, there have been no direct investigations of sensitivity to texture-defined information over a range of SFs and ages in childhood.

The current study therefore aimed to evaluate the development of contrast sensitivity to *both* luminance- and texture-defined information over a range of SFs in school-aged children. Children and adolescents are typically grouped together for simplicity and convenience; an approach that has influenced our understanding of the development profiles of luminance- and texture-defined information^37^. In our study, however, we grouped participants into discrete age bins to gain a better understanding of how sensitivities develop across age and SF during the school-age years. Different luminance- and texture-defined contrast sensitivity profiles across the school-age groups would suggest separate mechanisms underlying their respective perception during development.

## Methods

### Participants

Forty typically developing children and ten adults were recruited from an existing participant list and from advertisements in a community-based, family magazine. These participants were placed into five age groups: (1) 5–6 years (*n* = 10, mean age *M* = 5.8 years, *SD* = 0.57) ; (2) 7–8 years (*n* = 10, *M* = 7.87 years, *SD* = 0.69) ; (3) 9–10 years (*n* = 10, *M* = 9.97 years, *SD* = 0.55) ; (4) 11–12 years (*n* = 10, *M* = 11.77 years, *SD* = 0.47) ; (5) 18–35 years (*n* = 10, *M* = 24.4 years, *SD* = 4.86).

Before the testing procedure, all participants except the adults completed the Peabody Picture Vocabulary Test^38^ (PPVT-R; for English-speaking participants) or the Échelle de Vocabulaire en Images Peabody^39^ (EVIP; for French-speaking participants). The PPVT and EVIP are standardized tests used to evaluate verbal mental age. All participants scored well within the normal range for their age (5–6 years, mean verbal age *M* = 6.01, *SD* = 1.04; 7–8 years; *M* = 8.75, *SD* = 1.37; 9–10 years, *M* = 12.41, *SD* = 3.00; 11–12 years, *M* = 14.47; *SD* = 1.82), and were therefore considered to be developing typically.

Near and far point directional -C and -E cards (Logarithmic Visual Acuity Chart Landolt “C” and Tumbling “E” Folding Distance Chart; https://precision-vision.com/) were used to assess visual acuity and revealed that all participants had normal or corrected-to-normal visual acuity (i.e., ≥ 20/25 or 6/7.5). None of the participants had a history of visual problems or psychiatric or neurodevelopmental disorders (e.g., Attention Deficit Hyperactivity Disorder) according to self- or parental-report and all were inexperienced psychophysical observers. Parents or caregivers of the minor participants and adult participants provided written informed consent, following the ethical procedures and guidelines outlined by McGill University and the Declaration of Helsinki.

### Apparatus and stimuli

A MacPro G4 computer running the VPIXX graphics (vpixx.com) program was used to generate and present the stimuli. The luminance resolution produced by this apparatus was equivalent to an 11-bit video digital-to-analogue converter. A calibrated, 18-inch Viewsonic E90FB 0.25 CRT monitor (1,600 × 1,200 pixels) was used to present the stimuli and was refreshed at a rate of 75 Hz. The mean luminance of the display was set to 50 cd/m^2^, where L_min_ and L_max_ were 0.5 and 99.5 cd/m^2^, respectively. Gamma correction was verified at regular intervals using a 256 × 3 matrix color look-up table (CLUT) and a Minolta CS-100 Chroma Meter colorimeter. This procedure minimized the nonlinearities in the display to ensure that the texture-defined gratings were free of luminance artifacts.

**Figure 1:**
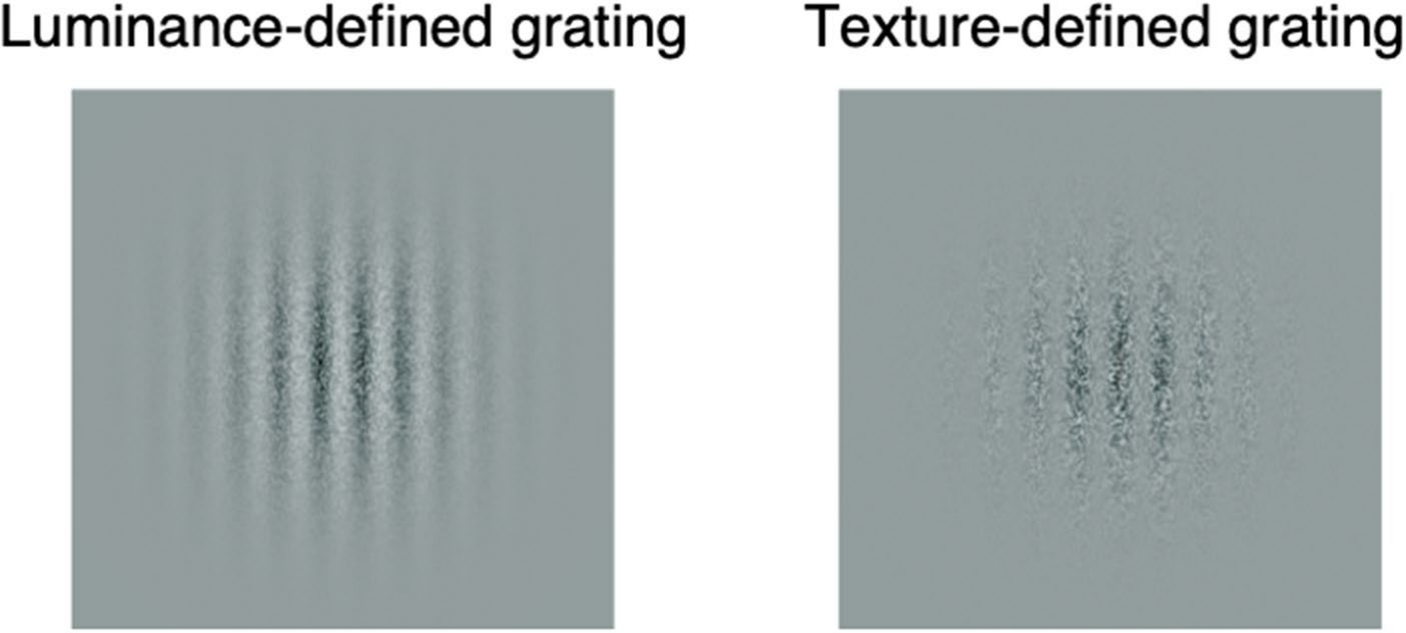
Stimuli samples of a luminance-defined grating (luminance modulated noise—left panel) and texture-defined grating (texture-contrast-modulated noise—right panel).

The luminance- and texture-defined stimuli used to measure spatial contrast sensitivity consisted of luminance- and texture-contrast-modulated sine-wave gratings, respectively (see Fig. 1). The gratings were multiplied with a circular Gaussian envelope (σ = 2°) and had a diameter of 10° when viewed from 57 cm. Both luminance- and texture-defined stimuli were constructed using a static, greyscale noise carrier with a mean luminance of 50 cd/m^2^, same as the mean luminance of the display. The carrier consisted of individual pixels measuring 2.235 arcmin, with individual pixel luminance levels randomly assigned as a function of sin(x), where (x) ranged from 0 to 2π and varied between 24.75 and 74.75 cd/m^2^ (or by half its maximum contrast^40^). New noise carriers were generated for each trial, and the initial phase of the modulating grating was set randomly.

Luminance-defined gratings were constructed by adding static greyscale noise to a modulating sinewave grating (luminance modulation), whereas the texture-defined gratings were constructed by multiplying the same carrier with the modulating sinewaves (contrast modulation). Mathematically, the luminance profile at point (x, y) of luminance (Eq. 1) and contrast-modulated (Eq. 2) gratings were defined as^41^:

1 $$l_{LM} \left( {x,y} \right) = L_{mean} \left[ {1 + \frac{{C_{c} }}{2}R\left( {x,y} \right) + \frac{C}{2}\sin \left( {2\pi \left( {f\left( {y\cos \theta - \sin \theta } \right) + \omega t} \right) + \phi } \right)} \right]$$

2 $$l_{CM} \left( {x,y} \right) = L_{mean} \left[ {1 + C_{c} R\left( {x,y} \right)\left( {1 + C\sin \left( {2\pi \left( {f\left( {y\cos \theta - \sin \theta } \right) + \omega t} \right) + \phi } \right)} \right)} \right]$$

where *L*_*mean*_ is the background luminance of the display, *R*(*x,y*) is the carrier, *C*_*c*_ is the contrast of the carrier, *f* is the SF of the modulating grating (luminance-defined gratings: 0.5, 1, 2, 4 and 8 cpd; texture-defined gratings: 0.5, 1, 2 and 4 cpd), *θ* is the orientation of the modulating sinewave (set to 0 deg, i.e., vertical gratings), *ω* is the temporal frequency of the modulating sinewave grating (set to 0 Hz, i.e., static gratings), *ϕ* is the initial spatial phase and *C* is the contrast of the envelope (luminance modulation: $\frac{{L_{max} - L_{min} }}{{L_{max} + L_{min} }}$, contrast modulation: $\frac{{C_{max} - C_{min} }}{{C_{max} + C_{min} }}$ ). Pilot testing revealed that the highest SF for texture-defined gratings (8 cpd) were not consistently visible for the youngest participants; texture-defined gratings of 8 cpd were therefore excluded from the actual task.

### Procedure

Participants sat comfortably 57 cm from the computer monitor in a quiet and dimly lit room. The experimenter began each session by explaining the task to the participant with both visual and verbal aids. Practice trials were completed before each experimental block (i.e., two blocks, luminance- and texture-defined gratings) with a 1 cpd grating, to familiarize the participant with the task before actual testing. Once the participant understood the task, the experimenter initiated the testing condition with the press of the spacebar on the keyboard. Participants maintained their gaze on the computer monitor and indicated in which of two spatial locations a target grating appeared. The task followed a two-alternative forced-choice procedure in which the center of a grating appeared to 6.5 degrees of visual angle to the left or right of the monitor’s center and unmodulated noise appeared on the opposite side. Gratings appeared for a maximum of 2 s. Children pointed to the target and the experimenter recorded their response. The experimenter encouraged short breaks after each SF condition to ensure that participants maintained their attention throughout the session.

Thresholds for each SF condition were recorded using a single adaptive staircase procedure (Harvey’s ML-PEST^42^). This staircase fitted a new psychometric function to the data after each trial and continued until the threshold estimate fell within ± 0.1 log units of the true threshold measure with 90% confidence. Contrast sensitivities were calculated by taking the inverse of the contrast detection thresholds for the luminance- and texture-defined gratings. These values were then used to plot contrast sensitivity functions. The running order of luminance- and texture-defined gratings was counterbalanced across participants. The entire testing session, including the receptive language and acuity assessments, lasted approximately one hour.

## Results

**Figure 2:**
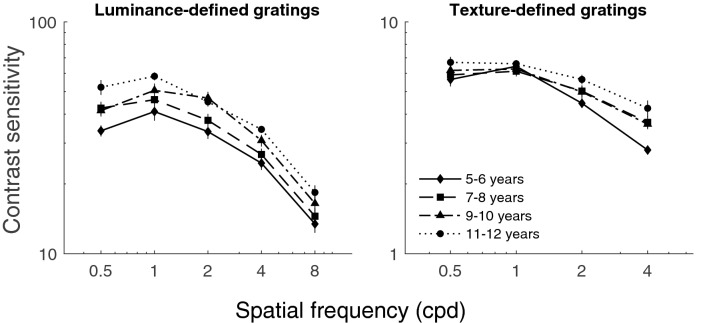
Contrast sensitivity of school-aged children. On the left, contrast sensitivity for luminance-defined gratings as a function of SF across age group is represented. On the right, contrast sensitivity for texture-defined gratings as a function of SF across age groups is represented. The error bars represent the standard error of the mean (some are not visible being smaller than the size of the marker). These graphs are represented on a log–log scale.

Contrast sensitivities for luminance- and texture-defined gratings are displayed in Fig. 2. For luminance-defined gratings, contrast sensitivity functions were band-pass with a peak at 1 cpd, and for the texture-defined gratings, they were low-pass with a cutoff at 1 cpd, which are in agreement with previous studies^43,44^. Separate analyses were subsequently conducted for contrast sensitivities in each condition: luminance- and texture-defined gratings. The gratings were based on different image attributes, meaning that absolute differences in the sensitivities were not meaningful^9,36^. Contrast sensitivities were therefore not compared for the different types of gratings.

A two-way ANOVA (age group x SF) for contrast sensitivities to luminance-defined gratings revealed a significant effect of SF, *F*_(4,144)_ = 307.2, *p* < 0.001, η_p_^2^ = 0.90, and age group, *F*_(3,36)_ = 6.84, *p* < 0.001, η_p_^2^ = 0.36. However, no significant interaction between age group and SF was found, *F*_(12,144)_ = 0.80, *p* = 0.65, η_p_^2^ = 0.06. These results suggested that, although younger observers were generally less sensitive to luminance-defined gratings, sensitivity for each age group changed with increasing SF in a similar manner.

Tukey’s post-hoc tests were used to explore the main effect of age by comparing the contrast sensitivity of each age group to the oldest age group, 11–12 year-olds. Post-hoc tests demonstrated that, compared to the 11–12 years children, 5–6 and 7–8 years children had significantly lower contrast sensitivities (5–6 years, *p* < 0.001 and 7–8 years, *p* < 0.05), suggesting that 9–10 years children had similar thresholds as the 11–12 year-olds for the luminance-defined gratings condition.

A two-way ANOVA (age group x SF) for contrast sensitivities to texture-defined stimuli showed a significant effect of SF, *F*_(3,108)_ = 136.6, *p* < 0.001, η_p_^2^ = 0.79, age group, *F*_(3,36)_ = 5.08, *p* < 0.01, η_p_^2^ = 0.30, as well as a significant interaction between age group and SF, *F*_(9, 108)_ = 2.15, *p* < 0.05, η_p_^2^ = 0.15. Tukey’s post-hoc comparisons for the main effect of age revealed that children of 5–6 years had significantly lower contrast sensitivity than those of 11–12 years, *p* < 0.01. To understand the interaction between SF and age group, Tukey’s post-hoc tests revealed that the 5–6 years children had significantly lower sensitivity to gratings of 4 cpd than the children of 11–12 years, *p* < 0.001. This result suggests that the sensitivity to texture-defined information develops differently as a function of SF in younger observers, defined by a selective decrease in sensitivity for higher-SF information.

**Figure 3:**
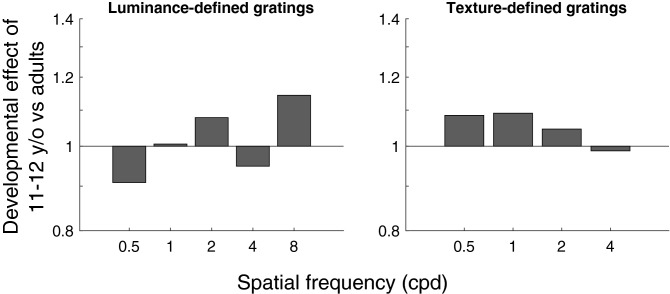
Developmental effect of 11–12 years old children versus a control, adult group. On the left, the developmental effect of the 11–12 years old children for each SF of the luminance-defined gratings condition. On the right, the same developmental effect for the texture-defined gratings condition.

A much older control group comprising 10 adults aged between 18 and 35 years was used to determine if the oldest children group (i.e., 11–12 years) reached visual maturity for the luminance and texture conditions. The developmental effect, defined by the ratio between the contrast sensitivity of the adult control group and the contrast sensitivity of the 11–12 years children at each SF for both conditions, are represented in Fig. 3. Overall, the developmental effect was small (< 1.14 factor) across SF and for both luminance- and texture-defined conditions. This effect was assessed statistically for each condition using a two-way ANOVA (age group x SF) comparing the sensitivity of 11–12 year-old group with that the adult control group. For the luminance condition, a significant effect of SF was found, *F*_(4,72)_ = 112.8, *p* < 0.001, η_p_^2^ = 0.86. However, no significant effect of age was found, *F*_(1,18)_ = 0.02, *p* = 0.89, η_p_^2^ = 0.001, nor was a significant SF x age group interaction, *F*_(4,72)_ = 1.12, *p* = 0.35, η_p_^2^ = 0.06. Similarly, a two-way ANOVA (age group x SF) for contrast sensitivities of texture-defined gratings of the 11–12 years children and adults revealed a significant effect of SF, *F*_(3,54)_ = 45.6, *p* < 0.001, η_p_^2^ = 0.72. However, no significant effect of age was found, *F*_(1,18)_ = 0.42, *p* = 0.52, η_p_^2^ = 0.02, and no significant interaction between age group and SF was found, *F*_(3,54)_ = 0.28, *p* = 0.84, η_p_^2^ = 0.02. These results suggest that 11–12 years children had adult-like thresholds across the whole range of SF for both luminance- and texture-defined gratings condition.

## Discussion

This study provides the first developmental account of contrast sensitivity to *both* luminance- and texture-defined gratings in the same sample of school-aged children. Using a range of SFs and ages, distinct contrast sensitivity functions were revealed for the luminance- and texture-defined gratings. The data capture subtle, but important, differences in sensitivities to information processed in early visual cortices.

For luminance-defined gratings, contrast sensitivity functions were band-pass across all age groups, with contrast sensitivity peaking at 1 cpd. This result is consistent with other studies that have added noise to their luminance-defined gratings^43,44^. In terms of development, the youngest children tested (5–6 and 7–8-year-old groups) had lower sensitivities to all SFs relative to the oldest children (i.e., 11–12 years). This suggests that sensitivity matures around the age of 9–10 years, and contrast sensitivity to luminance-defined gratings undergoes important developmental changes between the ages of 7–8 and 9–10 years. These results are consistent with previous studies reporting maturity of sensitivity to luminance-defined gratings at approximately 10 years of age^9,36^.

A similar development rate for sensitivity to luminance-defined gratings was found across SFs, suggesting that for each group, sensitivity changed in a similar manner as SFs increased. This finding differs from those of previous reports where there was a faster maturation for sensitivity to higher than lower SFs for luminance-defined information^45^. There are two possibilities for these discrepancies. First, unlike many other studies, we added noise to our luminance-defined gratings to equate them with the texture-defined gratings. The addition of background noise may have made the gratings slightly more difficult to detect and influenced the age at which maturity was reached. Second, we included four different age groups to examine development in a more detailed manner. Instead of using child and adult groups with wide age ranges, we used a smaller number of participants grouped into distinct age–bins of two years. This choice of grouping may explain why we did not find a specific interaction between age and SF for the luminance-defined gratings.

For the texture-defined condition, contrast sensitivity functions were low pass with a cutoff at 1 cpd across all age groups. This is consistent with previous studies conducted in children^9^ and adults^3,43^. Importantly, sensitivity to texture-defined information differed as a function of SF in younger observers, with a selective decrease in sensitivity for higher-SF information. These results point to the possibility that in the context of texture, the mechanisms mediating detailed information (i.e., high SFs) develop later than those mediating coarse information (i.e., low SFs). Future studies using similar stimuli and ages will be important for replicating this finding.

A noteworthy feature of this study is the different developmental profiles for the luminance and texture-defined gratings. Contrast sensitivity to luminance-defined gratings reached maturity in childhood at 9–10 years across all SFs, whereas contrast sensitivity to texture-defined gratings matured at 5–6 years for low SFs (i.e., 0.5 to 2 cpd), and 7–8 years for higher SFs (i.e., 4 cpd). Together, these results suggest that sensitivity matures earlier for texture- compared to luminance-defined information, consistent with previous studies^9,36^. One possibility for this difference may be that in adulthood, mechanisms mediating the processing of texture-defined information are less efficient than those mediating luminance-defined information^46,47^ (e.g., for texture-defined motion). Mechanisms mediating texture-defined information may not require as much refinement throughout development and therefore mature early in childhood.

Regardless of what mechanism underlies the difference in development across SF for the processing of texture-defined gratings, our results have clinical and practical implications. Many studies have shown that, relative to typically-developing peers, certain neurodevelopmental and pediatric patient populations show a decreased sensitivity or an atypical processing of texture-defined information^40,48–51^. For example, Bertone and colleagues^40,48^ noted important differences in how adults with Autism Spectrum Disorder (ASD) process luminance- and texture-defined information. Adults with ASD processed luminance-defined information better, but texture-defined information poorer, than typical adults. Others have extended these findings and reported that adolescents and adults with ASD demonstrate a lower sensitivity to texture-defined, circular forms compared to typical participants^52^. Together, these findings suggest that studying age-related changes to texture- rather than luminance-defined information may be a more sensitive and effective approach for evaluating visual profiles in both typical and atypical development^53,54^.

The current study has limitations. First, the participants did not undergo a full optometric screening. Though ideal, it is unlikely that this lack of screening affected our results, as all participants had normal or corrected-to-normal vision and were asked about their visual health. Second, the small sample size of each age group (i.e., n = 10) precluded a true developmental analysis, which would have yielded additional information in terms of differences in the onset and rates of development^55^. Third, our selection of SFs was limited in range, and it is possible that this affected our interpretation.

Despite these limitations, a major strength of this study is that it provides the first developmental account of spatial contrast sensitivity to *both* luminance- and texture-defined information. These findings demonstrate different developmental processes for luminance- and texture-defined information, with SF as an important factor when considering their developmental profiles.

## Data Availability

The data generated and analyzed during the current study are available from the corresponding author on reasonable request.
